# Influence of Various Fruit Preservation Methods on the Phenolic Composition and Antioxidant Activity of *Prunus spinosa* L. Fruit Extract

**DOI:** 10.3390/plants14152454

**Published:** 2025-08-07

**Authors:** Valentina Sallustio, Joana Marto, Lidia Maria Gonçalves, Manuela Mandrone, Ilaria Chiocchio, Michele Protti, Laura Mercolini, Barbara Luppi, Federica Bigucci, Angela Abruzzo, Teresa Cerchiara

**Affiliations:** 1 Drug Delivery Research Laboratory, Department of Pharmacy and Biotechnology, Alma Mater Studiorum, University of Bologna, Via San Donato 19/2, 40127 Bologna, Italy; valentina.sallustio2@unibo.it (V.S.); barbara.luppi@unibo.it (B.L.); federica.bigucci@unibo.it (F.B.); angela.abruzzo2@unibo.it (A.A.); 2 Research Institute for Medicines (iMed.ULisboa), Faculty of Pharmacy, Universidade de Lisboa, Avenida Professor Gama Pinto, 1649-038 Lisboa, Portugal; jmmarto@ff.ulisboa.pt (J.M.); lgoncalves@ff.ulisboa.pt (L.M.G.); 3 PharmaBot Laboratory, Department of Pharmacy and Biotechnology, Alma Mater Studiorum, University of Bologna, Via Irnerio 42, 40126 Bologna, Italy; manuela.mandrone2@unibo.it (M.M.); ilaria.chiocchio2@unibo.it (I.C.); 4 Pharmaco-Toxicological Analysis Laboratory (PTA Lab.), Department of Pharmacy and Biotechnology, Alma Mater Studiorum, University of Bologna, Via Belmeloro 6, 40126 Bologna, Italy; michele.protti2@unibo.it (M.P.); laura.mercolini@unibo.it (L.M.)

**Keywords:** *Prunus spinosa* L., preservation methods, antioxidant activity, hydroalcoholic extraction, phenolic compounds

## Abstract

Wild edible plants, historically valued for their medicinal properties, can be a sustainable source of food, cosmetics, and pharmaceuticals. The blue berries of *Prunus spinosa* L., known as blackthorns, have antioxidant, astringent, and antimicrobial benefits. To preserve these properties after harvesting, understanding the best storage methods is essential. In this study, blackthorns were preserved using different methods (air-drying, freezing, or freeze-drying) to determine the optimal procedure for preserving their antioxidant activity. The fruits were extracted using a 50:50 (*V*/*V*) mixture of ethanol and water. The different extracts were phytochemically characterized for their phenolic content and antioxidant activity. The Folin–Ciocalteu test revealed total phenolic contents of 7.97 ± 0.04, 13.99 ± 0.04, and 7.39 ± 0.08 (mg GAE/g raw material) for the three types of extracts, respectively. The total flavonoid contents were 2.42 ± 0.16, 3.14 ± 0.15, and 2.32 ± 0.03 (mg QE/g raw material), respectively. In line with the polyphenol analysis, the antioxidant activity as determined by DPPH method was higher for the frozen extract, with a value of 91.78 ± 0.80%, which was confirmed by the ROS test on keratinocytes. These results show that both air-drying and freeze-drying processes negatively impact the preservation of antioxidant activity in blackthorns, suggesting that freezing may be the best preservation method before bioactive compound extraction.

## 1. Introduction

In recent years, there has been a growing interest in the valorization of wild and underutilized plants as a sustainable resource that can contribute to the development of marginalized regions while simultaneously promoting biodiversity conservation [1]. Among the neglected and underutilized species, *Prunus spinosa* L. (PS) is becoming a more commonly investigated source for the food, cosmetic, and pharmaceutical industries, including all parts of the plant (leaves, flowers, fruits, stones) [2].

The plant species PS, characterized as a spiny shrub, is widely distributed in hilly and mountainous regions. This wild plant has significant potential for both organic agriculture and the preservation of biodiversity. It functions as a natural barrier that protects cultivated fields and offers a habitat for various wildlife species in organic farming settings. Furthermore, the roots of PS contribute to soil stabilization, while its abundant flowering enhances the habitat for pollinators, thereby playing a crucial role in ecosystem health [3].

The blue berries of PS, commonly known as blackthorns, are utilized in the production of various home-made products, including jams, syrups, and liqueurs [4]. All parts of the PS plant are traditionally used in medicine. The bark is used to prepare a decoction that helps reduce fever. An infusion of the leaves is utilized to alleviate constipation, while an infusion of the flowers is known to have digestive and laxative properties. Additionally, the fruits, which are rich in vitamin C, are beneficial for seasonal illnesses and aid in purifying the gastrointestinal tract [5].

Currently, blackthorns are being studied for their numerous benefits, which include antioxidant, anti-inflammatory, and antibacterial properties. These effects are linked to the variety of bioactive substances, more than 50 individual compounds, found in different parts of the blackthorn fruit: the kernel, pulp, and skin [6]. Notably, the skin of the blackthorn is rich in diverse types of polyphenols. Among these compounds, gallic acid and caffeic acid are examples of phenolic acids, while catechins and quercetin are classified as flavanols and flavonols, respectively [7]. Additionally, anthocyanins such as cyanidin 3-rutinoside and peonidin 3-rutinoside, responsible for the blue color of berries, have been explored in recent studies for their potential use as a natural food colorant [8,9,10].

These various phenolic compounds, combined with ascorbic acid in PS extract, exhibit significant antioxidant activity that is beneficial in preventing numerous diseases. However, many of these compounds are unstable when exposed to light and oxygen, which can diminish their antioxidant properties. Due to the small size of the fruits and the difficulty in harvesting them, as well as the plant’s often inaccessible growing locations, it is crucial to study effective conservation methods to preserve the activity of these functional substances [11]. Therefore, investigating post-harvest conservation methods is a fundamental step in designing an effective and sustainable food and pharmaceutical production chain.

Interestingly, the conservation of blackthorns is a surprisingly under-researched topic, especially when considering the limited use of this plant. Although the environmental benefits of the plant and health advantages of its fruits are well-documented, their excessive astringent taste restricts their consumption in raw form. To overcome this issue, the fruits are often processed into syrups, jams, jellies, or medicinal extracts [6,9,12,13]. This highlights that conservation is a critical factor in any potential transformation chain for these berries. Various methods can be employed to preserve wild berries, among which air-drying and hot-drying are the most prevalent techniques. These methods operate by removing moisture from the berries; however, the application of high temperatures may compromise the quality of the resultant product. Such thermal treatment can lead to the degradation of bioactive compounds and a decline in organoleptic properties, including changes in color (due to the thermal degradation of pigments), as well as alterations in taste and aroma resulting from the volatilization of essential oils [6]. In contrast, a properly conducted freezing process plays a crucial role in preserving the nutritional value and chemical composition of frozen berries, maintaining levels comparable to those found in fresh fruit while protecting thermolabile compounds [6,14]. Lyophilization, or freeze-drying, is distinguished by its ability to produce high-quality products with a prolonged shelf life, primarily through the inhibition of most chemical and microbiological reactions and a reduction in oxidation. Nonetheless, lyophilized products exhibit significant porosity and hygroscopicity, which may predispose them to oxidation and subsequent loss of valuable bioactive components [8].

Several previous studies have explored the preservation of blackthorn as an underutilized resource with significant potential in the food and pharmaceutical sectors. For example, Sikora et al. [15] compared the properties of fresh and frozen pulp, Magiera et al. [6] examined fresh and dried pulp, and Gunes et al. [16] analyzed dried and freeze-dried pulp. For the first time, we compared the three most commonly used preservation techniques applied to whole fruits instead of just the pulp. This approach may be beneficial for selecting a treatment that can be implemented immediately after harvest to maximize the time available for subsequent processing. For that purpose, in this study, we compared three preservation techniques for whole fruits: air-drying, freezing, and freeze-drying, with a specific focus on preserving antioxidant activity. We also considered sustainability, as each method has an increasing carbon footprint. We employed a hydroalcoholic solvent mixture along with ultrasound-assisted extraction, which has been identified as an effective and sustainable process to recover bioactive compounds [10,17,18]. Phytochemical analysis was conducted to evaluate the composition and antioxidant activity of the extracts. This activity was tested both using the DPPH assay and the ROS test. The three types of extracts were reanalyzed after 24 months to assess changes over time. Overall, this comprehensive comparison aims to identify the most effective and sustainable preservation method for maintaining the antioxidant properties of blackthorns, providing valuable insights for future applications in the food, nutraceutical, cosmetic, and pharmaceutical industries.

## 2. Results and Discussion

### 2.1. Morphological Description of Blackthorns

Fruits of PS named blackthorns were randomly collected from nearby plants in the same ripening stage. After harvesting, the main morphological characteristics (length, width, shape index, and weight) were measured, and the results are listed in Table 1.

The first step of this work involves measuring key parameters of the berries, such as diameter and shape, as these factors may influence the quantity of bioactive substances primarily found in the skin. Variability in phenotypes and pedoclimatic conditions affects the morphological characteristics of blackthorn, as noted by Cosmulescu et al. [3]. This study found a height range of 9.96 to 16.90 mm, a large diameter range of 9.62 to 17.09 mm, and a fruit weight between 0.90 and 3.90 g for different blackthorn phenotypes. Similarly, Oleińska et al. [19], in their research on blackthorns collected in Poland, reported a fruit length of 13.9 ± 1.50 mm, a width of 12.6 ± 1.13 mm, and a shape index of 1.10 ± 0.077. Additionally, Nistor et al. [12] indicated that the diameter of fruits from wild blackthorn plants is approximately 1 cm, while cultivated ones typically measure around 2 cm. The findings of our work align with the data reported by these authors.

### 2.2. Determination of Water Content of Blackthorns

Water content can influence the extraction yield, nutrient and non-nutrient concentrations, and practical issues such as handling properties, transportability, and storage of biowaste. The detected water content of 51.93 ± 1.66% is lower than the range reported by Gunes et al. [16], which spans from 60.86% to 69.37%. It is noted that this parameter can be influenced by the variety, pedoclimatic conditions, and ripening time [7,16].

### 2.3. Extraction by Bio-Solvents of Air-Dried (PSD), Frozen (PSF), and Lyophilized (PSL) Blackthorns

**Figure 1:**
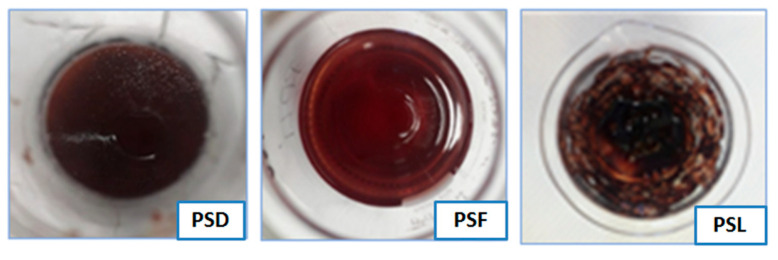
The appearance of extracts from blackthorns air-dried (PSD), frozen (PSF), and lyophilized (PSL).

A solution of ethanol:water 50:50 (*V*/*V*) and ultrasound-assisted extraction were used to extract the bioactive compounds from the vegetal matrix [20]. After concentrating the hydroalcoholic solutions, different extracts were obtained; their extraction yields are reported in Table 2, and they are shown in Figure 1.

In our work, we selected a mixture of ethanol, a green solvent, and water to obtain polyphenolic extracts from blackthorn fruits with enhanced antioxidant properties. Based on the literature, a 50:50 mixture of ethanol and water (*V*/*V*) at room temperature was used to achieve the optimal antioxidant activity. In fact, previous studies reported that a percentage of ethanol higher than 50%, as well as temperature values higher than 40 °C, can lead to a decrease in polyphenol extraction [21,22]. Ultrasound-assisted extraction (UAE), as an inexpensive, simple, and efficient technique, was used to improve the polyphenol extraction; this capability comes from mechanical and cavitation effects that disrupt cells, reduce the particle size, and enhance mass transfer across cell membranes [10].

The extraction yield for PSL was slightly higher than those of PSD and PSF, measuring 0.55 ± 0.14 g of extract per gram of raw material. However, the yields were generally consistent across all three types of extracts evaluated. It is expected that using air-dried or freeze-dried fruits can enhance the yield, as these forms are anhydrous. However, the extraction of polyphenols likely requires the swelling of the matrix, which occurs more slowly than in the frozen matrix. Therefore, achieving a higher yield from the dried matrix may require longer extraction times due to the need for rehydration and matrix swelling [23].

Regarding the visual appearance, it was observed that the PSF extract displayed a red–violet color, while the PSL extract and, more prominently, the PSD extract exhibited a brownish hue. Gunes et al. [8] demonstrated significant changes in the color parameters of air-dried and freeze-dried fruits when compared to fresh fruits, highlighting the notable effects of these drying processes. This color change, likely due to the exposure of the matrix to air, may indicate the oxidation of certain bioactive compounds, particularly anthocyanins, as reported by other authors in similar studies [9,24].

### 2.4. Analysis of Blackthorn Extracts

The main phytochemical analysis was performed on extract solutions at 1 mg/mL.

Regarding the pH values, all extract solutions displayed an acidic pH. Specifically, the pH values were 4.28 ± 0.06 for PSD, 4.33 ± 0.04 for PSF, and 4.34 ± 0.05 for PSL. These values are attributed to the presence of organic acids in the extracts, primarily malic, tartaric, succinic, aspartic, caffeic, and ascorbic acids [25,26].

The phytochemical characterization of blackthorn extracts unveiled different phenolic compounds with remarkable biological significance, with us highlighting their different potential through antioxidant activity assessment. In particular, the TPC and TFC were determined, and the results are reported in Table 3.

The TPC was determined spectrophotometrically using the Folin–Ciocalteu method, and gallic acid was used as the standard for the calibration curve (R^2^ = 0.999). TPC values are similar to those reported by Gunes et al. [16] and higher than those reported by Marcetic et al. [27]. Sikora et al. [15] reported a value for polyphenols from frozen blackthorn of 539.5 ± 17.7 mg/100 f.m. It is important to note that the authors preserved not the entire fruit but only the pulp. Additionally, in previous studies, different solvents were used, demonstrating that the choice of solvent can influence extraction parameters. In this study, ethanol was preferred over methanol; while methanol might be more effective, it is less sustainable than ethanol due to its toxicity [18,21]. Noticeably, the highest TPC was observed for the extract from frozen fruits, demonstrating that the preservation methods have a different influence on phenolic extraction of blackthorns. A study by Magiera et al. [6] investigated the evidence that blackthorn, when dried under conditions typical for herbal medicine production, contains lower levels of all types of polyphenols compared to fresh fruits. The authors noted that among the various compounds, anthocyanins and proanthocyanidins showed the most significant changes, with their total levels in the dried fruits decreasing by nearly 100% and 76%, respectively [6].

The TFC was determined spectrophotometrically with aluminum chloride as the reagent and quercetin as a standard for the calibration curve (R^2^ = 0.998). Similarly to the TPC, the value of TFC was higher for PSF (3.14 ± 0.15 mg QE/g RM) than for PSD and PSL. The main flavonoids identified for PS are rutin, quercetin, and myricetin [7]. It is important to note that some flavonoids, particularly rutin and quercetin, are especially thermolabile. Therefore, if a high quantity of flavonoids is desired, a lower temperature should be employed in preservation and extraction procedures [17].

Considering the results of the phytochemical analysis, which indicated that PSF is the most promising extract, to better characterize the composition, we performed FT-IR, ^1^H-NMR, and HPLC-DAD-MS/MS of this extract.

Moreover, TPC and TFC analysis was repeated after 24 months, and the values are reported in Table 3.

In terms of long-term stability, after storing all extracts at 4 ± 1 °C for 24 months, a decrease in TPC and TFC values was noted. However, the reduction was less evident for the PSF extract, highlighting it as the richest in phenolic compounds. Similarly, Oleińska et al. [19] compared lyophilized and frozen fruits and found that the TPC of lyophilized fruit was higher than that of frozen fruit. However, the authors reported that over time, the TPC and the ability to reduce iron ions increased in the frozen extract.

### 2.5. FT-IR of PSF Extract

**Figure 2:**
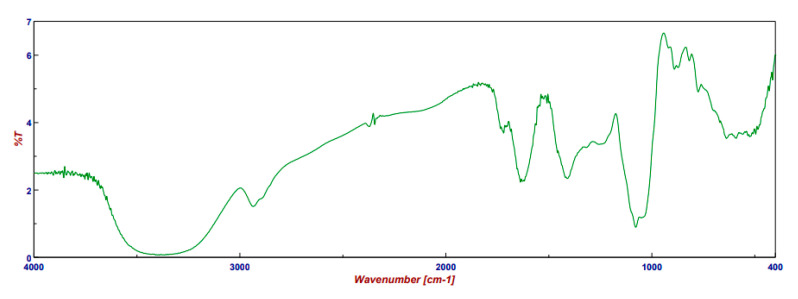
FT-IR spectrum of PSF extract.

The FT-IR spectrum of PSF, noted for having the highest TPC and TFC values, and exhibiting great stability, is presented in Figure 2, which can be referenced to identify the main functional groups of the chemical compounds.

In Figure 2, an intense band at 3380 cm^−1^ is attributed to polyphenols [28]. The oscillation of the carbonyl groups in fructose and glucose is observed in the range of 1750–1550 cm^−1^. Absorption bands for the carboxyl and ester groups can be found between 1740 and 1720 cm^−1^ [29]. The band at 1200–1000 cm^−1^ is associated with the stretching vibrations of the (C-OH) side groups and the glycosidic bond (C-O-C) vibrations within polysaccharide chains. Bands in the range of 890–760 cm^−1^ correlate with the specific oscillations in the anomeric region of carbohydrates, or C-H deformation [30]. Additionally, absorption peaks around 520 cm^−1^ are related to the presence of anthocyanin pigments [28]. The FT-IR spectrum profile is consistent with the FT-IR analysis of extracts reported in the literature [11,14,28].

### 2.6. ^1^H-NMR Analysis of PSF Extract

The PSF metabolite composition was characterized through ^1^H-NMR profiling, which is commonly employed for the rapid identification and semi-quantification of the most abundant metabolites.

**Figure 3:**
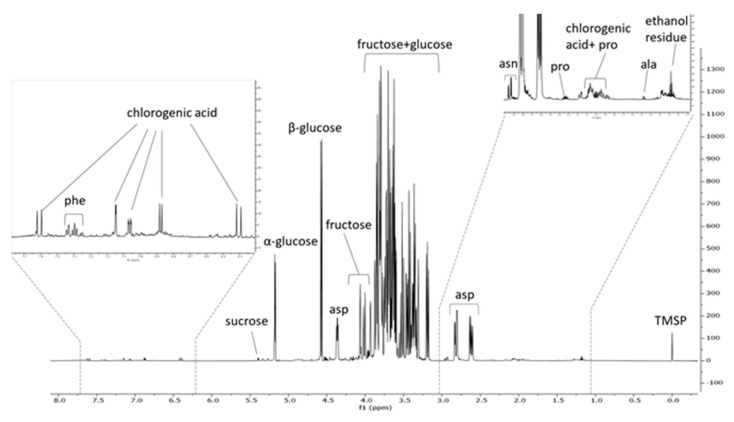
^1^H-NMR spectrum of PSF extract. TMSP = internal standard (trimethylsilyl propionic acid sodium salt). ala = alanine; asn = asparagine; asp = aspartic acid; phe = phenylalanine; pro = proline.

As shown in Figure 3, PSF mainly comprised primary metabolites, the most abundant being glucose, as highlighted by the presence of the doublets at δ 5.2 and δ 4.6 ascribable to the anomeric protons of α and β glucose, respectively, and aspartic acid. Fructose was also abundant, with a semi-quantified content of 150.2 μg/mg (RM) (Table 4). In the aromatic region (between δ 8 and 6 approximately), the amino acid phenylalanine and the phenolic compound chlorogenic acid were detected. Hence, this analysis allowed us to identify chlorogenic acid as the most abundant specialized metabolite in the extract as well as to explain approximately 80% (*w*/*w*) of the extract composition.

### 2.7. HPLC-DAD-MS/MS Analysis of PSF Extract

Since antioxidant activity is the main focus of this study, the most stable extract (PSF) was further analyzed to quantitatively investigate representative classes of antioxidant compounds. The HPLC-DAD-MS/MS analysis of PSF provided an overview of some representative phenolic compounds and ascorbic acid; their values are shown in Table 5.

Among the phenolic compounds analyzed, catechin is the most abundant, followed by cyanidin and quercetin. According to the literature, flavonoids and anthocyanins accounted for approximately one-tenth of the polyphenols in blackthorn fruits. Other phenolic acids detected in the extract were caffeoyl-, coumaroyl-, and feruloylquinic acids; among flavonoids, there were mostly quercetin mono-, di-, and triglycosides; and finally, among anthocyanins, there were cyanidin and peonidin glycosides [14]. In addition, vitamin C was measured at a level of 1.08 ± 0.34 mg/g in the raw frozen material, which is higher than the 23.84 mg/100 g f.m. reported by Sikora et al. [15]. Vitamin C is a major contributor to the antioxidant activity of blackthorn extract [7], as shown in the following sections.

### 2.8. Determination of Antioxidant Activity (AA%)

The antioxidant activity is predictive of many biological benefits of natural compounds. In this work, two methods were chosen to determine the antioxidant activity. The first method was the chemical DPPH assay, and the second was the biological determination of ROS reduction using the HaCaT cell line.

#### 2.8.1. Determination of AA% by DPPH

**Figure 4:**
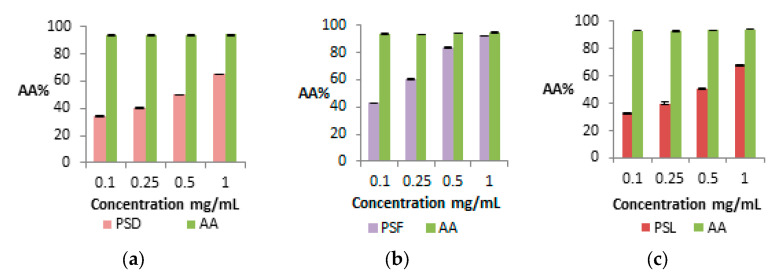
Antioxidant activity of blackthorn extracts by DPPH versus ascorbic acid (AA) (green column). (**a**) PSD (rose column), (**b**) PSF (violet column), (**c**) PSL (red column). The data are expressed as the mean of three replicate experiments ± SD.

The antioxidant activity of DPPH for different extract concentrations (0.1, 0.25, 0.5, and 1 mg/mL) was assessed in comparison with a standard solution of ascorbic acid as a reference substance. The results are shown in Figure 4.

The analysis of the DPPH assay at different concentrations of the extracts shows that the AA% is dose-dependent between 0.1 and 1 mg/mL. This confirms that the AA% of PSF is higher than those of PSD and PSL across all tested concentrations. The IC_50_ values were also calculated, coming in at 0.543 mg/mL, 0.139 mg/mL, and 0.512 mg/mL for PSD, PSF, and PSL, respectively. These results align with the TPC and TFC values of the extracts.

Phenolic compounds are commonly regarded as highly unstable and prone to degradation. Therefore, understanding the stability of polyphenols under various conditions is crucial. This knowledge is essential to ensure that the final product retains its desired properties while maintaining the biological activity and structural integrity of the compounds throughout the different stages of processing [17].

The stability of standard polyphenols and plant extracts against UV irradiation is relatively high, with the highest stability observed for gallic and vanillic acids. In contrast, anthocyanins are easily oxidized, making them susceptible to oxidative degradation during various stages of processing and storage. Several factors influence the stability of anthocyanins and, consequently, the products that contain them. These factors include pH, temperature, light, and oxygen [24]. In particular, concerning anthocyanins, the oxidative polymerization is intensified during thermal fruit preservation in long-stored products under non-refrigerated conditions; an irreversible oxidation of anthocyanin pigments can be visually observed as a color change from red–violet to red–brown [9,19,31,32]. Focusing on flavonoids, another aspect that decreases the antioxidant activity in air-dried or freeze-dried fruits is that these preservation methods lead to greater aeration of powdered blackthorns, which contributes to the oxidation of these bioactive compounds. In contrast, the aeration of pulp is limited when using frozen fruits, and Sikora et al. [15] and Marcetic et al. [27] have reported that the differences between frozen and fresh fruits are not statistically significant. Moreover, vitamins such as ascorbic acid in the blackthorn contribute to antioxidant activity; however, preservation processes such as drying or air exposure could oxidize vitamin C. For this reason, PSF antioxidant activity could also be high due to the presence of the active form of vitamin C. According to the literature, the antioxidant activity of different preserved blackthorns depends not only their phenolic and vitamin composition but also on the oxidative process related to the temperature and the duration of storage [6,12,15,16].

To better understand whether the differences in the antioxidant activity of extracts were maintained over time, the DPPH assay was performed again after 24 months of extract storage at 4 ± 1 °C.

Table 6 presents the comparison of the DPPH assay values, showing that PSF demonstrated the highest AA% even after the extract had been stored for 24 months. This finding is consistent with the results from the TPC and TFC determinations. Therefore, we can conclude that for PSF, a higher phenolic content correlates with greater antioxidant activity, which is notably maintained over time.

#### 2.8.2. Antioxidant Activity by ROS Determination of PSF Extract 

**Figure 5:**
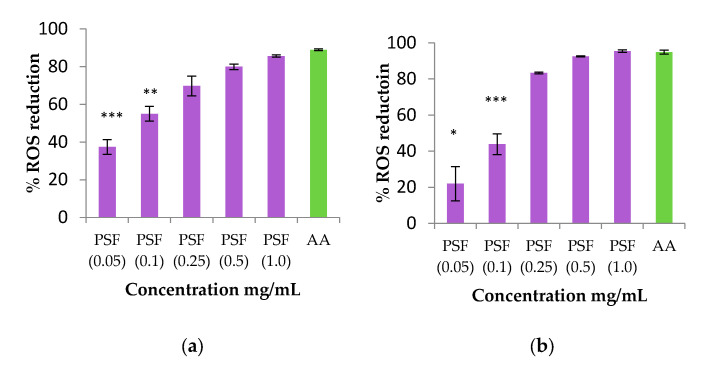
Reactive oxygen species reduction produced by blackthorn extract solution (violet column) at different concentrations after exposure to H_2_O_2_ and UVB radiation of HaCaT cells. The data are expressed as the mean of at least nine replicate experiments ± SD. Significance: (*) *p* < 0.05, (**) *p* < 0.01, (***) *p* < 0.001 versus positive control cells (ascorbic acid, green column). (**a**) H_2_O_2_. (**b**) UV.

Based on the results of the DPPH test, PSF was identified as the most effective for the antioxidant activity as chemically determined. In addition, the antioxidant activity of the PSF extract was further evaluated through a biological assay measuring its capacity to reduce intracellular ROS induced both by a chemical compound (H_2_O_2_) or by UV light in vitro in HaCaT (human keratinocyte cell line). The results are shown in Figure 5.

According to the DPPH assay, a similar trend was found for blackthorn extract in the ROS reduction assay. The IC_50_ was also determined after exposure to H_2_O_2_ and UVB radiation, with values of 75.6 ± 1.2 and 115.0 ± 1.0 µg/mL, respectively.

Taking into consideration all analysis results, the PSF extract showed the best characteristics and preservation of biological activity and, for this reason, it could be selected as a functional ingredient for food, cosmetic, and pharmaceutical products.

## 3. Materials and Methods

### 3.1. Chemicals

Folin–Ciocalteu reagent was sourced from Titolchimica (Pontecchio Polesine, Italy). Gallic acid, quercetin, cyanidin, catechin, and ascorbic acid were purchased from Sigma Aldrich (Milan, Italy). Deuterium oxide (D_2_O, 99.90% D) and CD_3_OD (99.80% D) were purchased from Eurisotop (Cambridge Isotope Laboratories, Inc., Saint-Aubin, France). Standard 3-(trimethylsilyl)-propionic-2,2,3,3-d4 acid sodium salt (TMSP), sodium phosphate dibasic anhydrous and sodium phosphate monobasic anhydrous, and Dulbecco’s modified Eagle medium supplemented with D-glucose were purchased from Sigma-Aldrich Co. (St. Louis, MO, USA). Fetal bovine serum (FBS), L-glutamine, penicillin (1000 U/mL), and streptomycin were purchased from Euroclone S.p.A. (Milan, Italy). All solvents and other chemicals were purchased from Sigma-Aldrich (Milan, Italy). Ultrapure water (18.2 MΩ cm) was obtained with the MilliQ apparatus by Millipore (Milford, MA, USA).

### 3.2. Plant Materials

Fruits of *Prunus spinosa* L. (blackthorns) spontaneously growing were randomly collected in November 2020 in their proper ripening stage to assure the presence of a high amount of polyphenols in rural areas of Brisighella (Emilia Romagna, Italy), GPS coordinates 44°12′46.9” N 11°50′46.6” E. A batch of fruits was immediately analyzed for morphological description and water content, while other batches were preserved as detailed in Section 3.5.

### 3.3. Morphological Description

The main morphological characteristics (length, width, shape index, weight) were determined for 40 samples of fruits. A fruit’s length (L) was measured with an electronic digital caliper (art. 1367 E 2900, Shanghai ShangErBo Import & Export Co., Shanghai, China). The fruit’s width (W) was determined at the widest part perpendicular to its length. The fruit shape index was calculated as the length-to-width ratio (L/W). The weight of the fruit was measured by means of an analytical balance (ED22DS Sartorius, Göttingen, Germany).

### 3.4. Determination of Water Content

The water content was determined by weighing 20 g of blackthorns, placing them on a watch glass, and oven-drying (heating oven FD series; Binder, Tuttlingen, Germany) at 105.0 ± 2.0 °C for 48 h until a constant mass was achieved. After this period, the samples were weighed again to calculate the water percentage. The determination was performed in triplicate.

### 3.5. Fruit Preservation Methods

**Figure 6:**
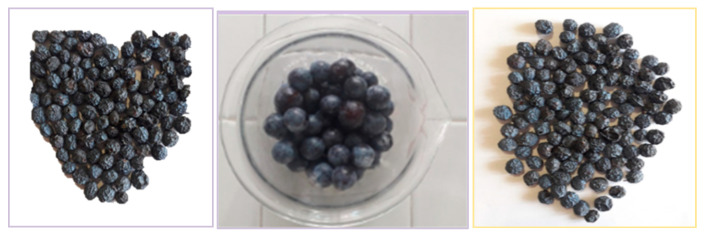
Pictures of blackthorn air dried (PSD), frozen (PSF), and lyophilized (PSL).

Immediately after harvesting, blackthorns were gently cleaned and stored by using three different methods of preservation: freezing, air-drying, and lyophilization. Freezing was performed in a commercial freezer at −20 °C, air-drying was performed in the dark at room temperature for 20 days, and lyophilization was performed with a vacuum freeze-dryer (Christ Freeze Dryer ALPHA 1-2, Milan, Italy) at 0.01 atm and −48 °C for 72 h (Figure 6). The fruits were stored in the dark until the extraction process, which was performed 12 weeks after ripening.

### 3.6. Extraction of Blackthorns by Bio-Solvents

**Figure 7:**
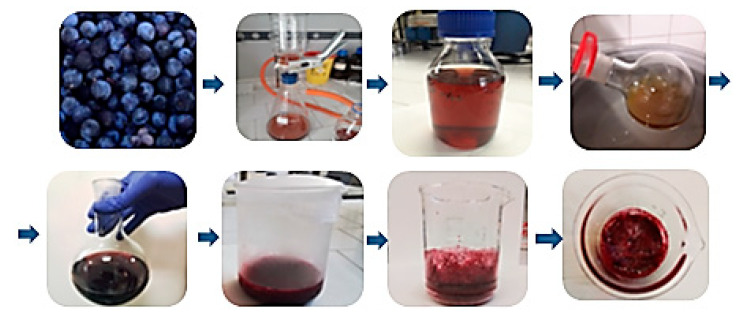
Steps of blackthorn extraction.

For the hydroalcoholic extraction, the fruit stones were carefully removed using a thin knife, and the pulp was homogenized by using a laboratory blender. Then, 10 g of the pulp was mixed with 150 mL of a 50:50 *(V/V)* ethanol:water solution and sonicated (Transonic TP690 by Elma, Singen, Germany) at room temperature for 40 min. After sonication, the mixture was centrifuged at 4000 rpm for 20 min, and the supernatant was filtered using a Buchner filter (Rotofix 32A by Hettich, Tuttlingen, Germany). To increase the extraction yield, the residue was subjected to further extraction by repeating the procedure three additional times. The collected supernatants were then evaporated under vacuum at 40 °C (Buchi Rotavapor Heating Bath B-490, Flawil, Switzerland) to eliminate the ethanol and most of the water. The concentrated extracts were lyophilized at −48 °C for 48 h and stored at 4.0 ± 1.0 °C in the dark until ready for use (Figure 7).

### 3.7. Analysis of Extracts

#### 3.7.1. Determination of the pH

The pH value of extract solutions was determined by using a digital pH meter (Crison Instruments, S.A., Barcelona, Spain). The glass electrode was calibrated with the solutions determined for the equipment (pH of 4.00 and 7.00). The analysis of the pH of each solution was performed in triplicate, and average values were calculated.

#### 3.7.2. Determination of Total Phenolic Content (TPC)

The TPC was determined using the Folin–Ciocalteu reagent, following the method described by Singleton et al. [33]. This analysis was performed for extracts freshly prepared and again after 24 months of storage at 4 ± 1 °C. In brief, 0.2 mL of an extract solution (1 mg/mL) was mixed with 1.00 mL of the Folin–Ciocalteu phenol reagent diluted 1:10, followed by the addition of 0.8 mL of sodium carbonate solution (7.5% *w*/*V*). The mixture was then incubated in the dark for 30 min at a temperature of 40.0 ± 1.0 °C. The absorbance was measured spectrophotometrically at 750 nm using a UV-Vis 1601 spectrophotometer (Shimadzu, Milan, Italy), with distilled water serving as the blank. TPC was calculated based on a standard curve of gallic acid in the 0–0.25 mg/mL concentration range (R^2^ = 0.999). All measurements were conducted in triplicate, and the results were expressed as gallic acid equivalents in milligrams per gram of raw material (mg GAE/g RM).

#### 3.7.3. Determination of Total Flavonoid Content (TFC)

The TFC was determined using a modified method from the European Pharmacopeia [34]. This analysis was performed for extracts freshly prepared and again after 24 months of storage at 4 ± 1 °C. In brief, 1 mL of a 2% (*w*/*V*) aluminum chloride (AlCl_3_) solution was added to 1 mL of the extract solution (1 mg/mL). The mixture was then incubated at room temperature in the dark for 30 min, after which the absorbance was measured at 430 nm. The TFC was calculated based on a standard curve using quercetin in the 0–0.01 mg/mL concentration range (R^2^ = 0.999) and is expressed as milligrams of quercetin equivalent per gram of raw material (mg QE/g RM).

### 3.8. FT-IR of PSF Extract

The FT-IR spectrum was obtained using a Jasco FT-IR-4100 spectrophotometer (Jasco, Lecco, Italy) with the KBr pellet technique. The sample was prepared by mixing the extract with KBr in a ratio of 1:9 and then compressing the mixture under pressure. Measurements were conducted over an infrared range of 400 to 4000 cm^−1^.

### 3.9. ^1^H-NMR of PSF Extract

The selected extract was solubilized at a concentration of 310 mg/mL in a blend of solvents comprising phosphate buffer (90 mM; pH 6.0) in H_2_O-d_2_ (containing 0.01% TMSP) and MeOH-d_4_ (1:1) and subjected to ^1^H-NMR analysis. The spectrum was recorded at 25 °C on a Varian Inova instrument (equipped with a reverse triple resonance probe) operating at a ^1^H-NMR frequency of 600.13 MHz, and MeOH-d_4_ was used as the internal lock. The spectrum consisted of 256 scans (corresponding to 16 min) with the relaxation delay (RD) of 2 s, acquisition time 0.707 s, and spectral width of 9595.8 Hz (corresponding to δ 16.0). A presaturation sequence (PRESAT) was used to suppress the residual water signal at δ 4.83 (power = −6 dB, presaturation delay 2 s). The spectrum was manually phased, baseline corrected, and calibrated to the internal standard TMSP at δ 0.0 using Mestrenova software (Mestrelab Research, Santiago de Compostela, Spain). TMSP was also used to semi-quantify the identified metabolites in the extract.

### 3.10. HPLC-DAD-MS/MS of PSF Extract

The analytical evaluation of PSF was carried out using a previously developed and validated HPLC-DAD-MS/MS method specifically designed for the quantification of plant-derived phenolic compounds and ascorbic acid [34]. The system setup included a Waters Alliance e2695 HPLC (Milford, MA, USA) coupled with a Waters 2998 diode array detector and interfaced with a Micromass Quattro Micro triple quadrupole mass spectrometer. For chromatographic separation, a reversed-phase Restek Ultra AQ C18 column (dimensions: 50 × 2.1 mm, particle size: 3 µm) was used. The mass spectrometer was operated with electrospray ionization in the negative mode (ESI–), and compound detection was performed through multiple reaction monitoring (MRM). Given the differing polarity and chemical behavior of the analytes, two distinct analytical workflows were applied: one for polyphenolic markers (cyanidin, gallic acid, catechin, and quercetin), and a second for vitamin C (ascorbic acid).

For the analysis of phenolic compounds, the mobile phase was composed of (A) acetonitrile containing 0.1% formic acid (*V*/*V*) and (B) ultrapure water also acidified with 0.1% formic acid (*V*/*V*). The gradient profile was structured as follows: starting at 2% A and held steady for 3 min, then linearly increased to 15% A over 2 min, kept isocratic for 4 min, followed by a gradual return to 2% A in 5 min, and a final re-equilibration step at 2% A for 2 min. The flow rate was maintained at 0.4 mL/min. The injection volume was 10 µL. The MS source was configured with the following settings: capillary voltage 3.0 kV, source temperature 130 °C, desolvation temperature 350 °C, desolvation gas (nitrogen) flow at 600 L/h, and scan duration of 0.3 s. Argon was used as the collision gas. Each phenolic compound was quantified via the following MRM transitions and parameters: cyanidin: *m*/*z* 287.11 → 121.2, cone voltage 20 V, collision energy 15 eV; gallic acid: *m*/*z* 168.89 → 124.9, cone voltage 26 V, collision energy 10 eV; (+)-catechin: *m*/*z* 291.01 → 138.9, cone voltage 20 V, collision energy 14 eV; quercetin: *m*/*z* 301.02 → 150.9, cone voltage 27 V, collision energy 22 eV. Simultaneously, UV-DAD detection was conducted across the 200–400 nm range. For quantitation, the following wavelengths were selected based on compound-specific absorbance maxima: 285 nm for cyanidin, 270 nm for gallic acid, 260 nm for catechin, and 370 nm for quercetin.

For the determination of ascorbic acid, an isocratic method was employed using a mobile phase consisting of methanol and ultrapure water (15:85, *V*/*V*), both acidified with 0.1% formic acid (*V*/*V*). The flow rate was set at 0.3 mL/min, and the same C18 column described above was used. The MS parameters were adjusted accordingly: capillary voltage of 4.0 kV, source temperature of 115 °C, desolvation temperature of 300 °C, desolvation gas (nitrogen) flow at 300 L/h, scan time of 0.3 s, and argon as collision gas. The monitored MRM transition for ascorbic acid was *m*/*z* 175.13 → 115.2, cone voltage 20 V, and collision energy 15 eV. UV-DAD detection for ascorbic acid was carried out at 251 nm. The injection volume was also 10 µL. Quantification was performed by external calibration using analytical standards of gallic acid, catechin, quercetin, cyanidin, and ascorbic acid. The calibration curves were prepared in triplicate over a concentration range of 0.1–50 µg/mL and yielded determination coefficients (R^2^) greater than 0.999 for all analytes. The method provided limits of detection (LODs) in the range of 0.015–0.03 µg/mL and limits of quantification (LOQs) between 0.05 and 0.1 µg/mL, depending on the compound. These parameters, as well as intra- and inter-day precision (RSD < 7.8%) and extraction yield (>87%), were fully validated.

### 3.11. Antioxidant Activity Determined by DPPH

The AA% was assessed using the 2,2-diphenyl-1-picrylhydrazyl (DPPH) radical reduction assay, following the method described by Brand-Williams et al. [35] with minor modifications. In this assay, 1 mL of various concentrations (0.1, 0.25, 0.5, and 1.0 mg/mL) of each extract, along with ascorbic acid (used as the standard antioxidant compound), were mixed with 1 mL of a DPPH solution (0.1 mM in methanol) at room temperature. The mixtures were kept in the dark for 30 min, after which the absorbance was measured at 517 nm. Methanol served as the blank solution, and the DPPH solution acted as the control. The results are expressed as the percentage of inhibition of the DPPH radical, calculated using the following equation: Inhibition% = [(A_0_ − A)/A_0_], where A_0_ represents the absorbance of the DPPH control and A denotes the absorbance of the sample with DPPH. Moreover, the IC_50_ (half maximal inhibitory concentration) was calculated. This parameter represents the concentration of a substance necessary to reduce 50% of DPPH free radical activity. This measurement is commonly used to assess the antioxidant capacity of substances, with a lower IC_50_ indicative of greater antioxidant potency. For the highest concentration of extracts (1 mg/mL), the DPPH assay was performed again after 24 months of storage at 4 ± 1 °C.

### 3.12. Antioxidant Activity by Reduction Oxygen Species (ROS) Production Measurement

The intracellular production of ROS within cells was assessed with a fluorimetric technique using 2′,7′-dichlorodihydrofluorescein diacetate (H_2_-DCFDA, Life Technologies, Horsham, UK). HaCaT (human keratinocyte cell line, CLS Cell Lines Service GmbH, Eppelheim, Germany) sub-confluent cells grown in 96-well plates were incubated for 30 min with 20 μM of H_2_-DCFDA in the dark at 37 °C. Medium was removed and fresh medium added to the cells before they were exposed to different concentrations of samples (0.05–1 mg/mL) and ascorbic acid (AA, 1 mg/mL) for 1.5 h. Hydrogen peroxide (H_2_O_2_) solution (500 μM, 1 h) or exposure to UVB light (emission wavelength 312 nm) for 15 min was used for the induction of ROS in cells. After exposure, ROS levels were determined at excitation 485 nm and emission 520 nm wavelengths using a fluorescence microplate reader (FLUOstar BMGLabtech, Ortenberg, Germany). Data from 9 replicates are reported as the percentage of ROS reduction, determined as 100-(fluorescence of exposed cells/fluorescence of unexposed control from the same experiment) × 100. The concentration of the samples that reduces 50% of ROS (IC_50_) was determined by non-linear regression analysis with GraphPad PRISM^®^ 5 software (San Diego, CA, USA, www.graphpad.com (accessed on 1 June 2025).

### 3.13. Statistical Analysis

The results are expressed in terms of the average of a minimum of three determinations followed by standard deviations (SD). Student’s *t*-test was used to determine whether there was a significant difference between groups. One-way analysis of variance (ANOVA) was used to determine whether there was a significant difference between datasets. *p*-values below 0.05 were considered statistically significant.

## 4. Conclusions

Choosing the best storage methods is essential to maintain the multifunctional activity of wild edible plants after harvesting.

In our study, we compared the content of bioactive compounds and the associated antioxidant activity of extracts obtained from three types of preserved blackthorns (air-dried, frozen, freeze-dried). In comparison to previous studies, we preserved the entire fruit, considering that processing could not occur close to the harvesting site. We found that the total phenolic content (TPC) and total flavonoid content (TFC) were higher in extracts from frozen blackthorn compared to those from air-dried and freeze-dried blackthorn. This observation was supported by the DPPH test, which showed that the antioxidant activity was dose-dependent and significantly greater for the frozen blackthorn extract. Furthermore, the antioxidant effects were confirmed by the reduction of reactive oxygen species (ROS). In conclusion, among various preservation techniques for blackthorn, our findings suggest that freezing the fruits produces extracts rich in polyphenols and is the most effective method for preserving their antioxidant activity.

## Figures and Tables

**Table 1:** Length (mm), width (mm), shape index, and weight (g) of fresh blackthorns *.

Sample	Length (mm)	Width (mm)	Shape Index	Weight (g)
Blackthorn	12.11 ± 0.88	11.98 ± 0.91	1.07 ± 0.03	1.30 ± 0.25

* Values are expressed as mean ± SD (*n* = 40).

**Table 2:** Yield of extraction (g extract/g raw material) of blackthorns air-dried (PSD), frozen (PSF), and lyophilized (PSL) *.

Sample	PSD	PSF	PSL
Yield (g extract/g raw material)	0.52 ± 0.08	0.51 ± 0.28	0.55 ± 0.14

* Values are expressed as mean ± SD (*n* = 3).

**Table 3:** TPC and TFC of blackthorn extracts and after 24 months of storage at 4 ± 1 °C.

Type of Extract	TPC (mg GAE/gRM) *	TPC (mg GAE/gRM) * 24 Months	TFC (mg QE/gRM) **	TFC (mg QE/gRM) ** 24 Months
PSD	7.97 ± 0.04 ^A^	3.71 ± 0.03 ^A^	2.42 ± 0.16 ^A^	1.27 ± 0.08 ^A^
PSF	13.99 ± 0.04 ^B^	9.90 ± 0.19 ^B^	3.14 ± 0.15 ^B^	1.61 ± 0.07 ^B^
PSL	7.39 ± 0.08 ^C^	4.02 ± 0.06 ^C^	2.32 ± 0.03 ^A^	1.29 ± 0.02 ^A^

PSD = extract from blackthorns air-dried; PSF = extract from blackthorns frozen, and PSL = extract from blackthorns lyophilized. * mg GAE/g RM: mg gallic acid equivalent/g raw material; ** mg QE/g RM: mg quercetin equivalent/g raw material. There is no statistical difference between the results shown with the same superscript capital letter in the same column (*p* > 0.05). Values are expressed as mean ± SD (*n* = 3).

**Table 4:** Semi-quantification of metabolites detected in PSF extract by means of ^1^H-NMR.

Metabolite	δ	Multiplicity	μg/mg PSF Extract	Percentage
**Alanine**	1.49	d	0.2	0.02
**Asparagine**	2.94	dd	3.8	0.4
**Aspartic acid**	2.64	dd	152.1	15.2
**Chlorogenic acid**	6.37	d	9.9	1
**Fructose**	3.98	m	150.2	15
**Phenylalanine**	7.37	m	1.3	0.1
**Proline**	2.35	m	2.6	0.3
**Sucrose**	5.4	d	10.8	1.1
**α glucose**	5.2	d	172.7	17.3
**β glucose**	4.6	d	288.3	28.8

d = doublet, dd = double doublet, m = multiplet.

**Table 5:** Content of phenolics and ascorbic acid of PSF *.

	Phenolic Acid	Flavanol	Flavonol	Anthocyanin	Vitamin
	Gallic Acid (mg/g RM)	Catechin (mg/g RM)	Quercetin (mg/g RM)	Cyanidin (mg/g RM)	Ascorbic Acid (mg/g RM)
PSF	0.18 ± 0.07	5.96 ± 0.74	1.46 ± 0.22	1.77 ± 0.32	1.08 ± 0.34

* Values are expressed as mean ± SD (*n* = 3).

**Table 6:** Antioxidant activity of blackthorn extract solutions (1 mg/mL) *.

Sample	PSD	PSF	PSL
AA%	65.02 ± 0.22 ^A^	91.78 ± 0.80 ^A^	68.39 ± 0.35 ^A^
AA% 24 months	37.79 ± 1.80 ^B^	81.57 ± 1.14 ^B^	29.79 ± 0.46 ^B^

* There is no statistical difference between the results shown with the same superscript capital letter in the same column (*p* > 0.05). Values are expressed as mean ± SD (*n* = 3).

## Data Availability

Data will be available from the authors on request.
